# Nursing, Commitment, and Leadership: More Nurses for a Better Health Care Model—Be a Nurse to Be a Leader

**DOI:** 10.3390/ijerph19106223

**Published:** 2022-05-20

**Authors:** Vicente Gea-Caballero, Patricia Marín-Maicas, Teresa Sufrate-Sorzano, Marco Di Nitto, Anna Rozensztrauch, Raúl Juárez-Vela

**Affiliations:** 1 Faculty of Health Sciences, International University of Valencia, 46002 Valencia, Spain; patricia.marin.m@campusviu.com; 2 Research Group Nursing and Community Health SALCOM, International University of Valencia, 46002 Valencia, Spain; 3 Research Group in Care, School of Nursing, University of La Rioja, 26004 Logroño, Spain; teresa.sufrate@unirioja.es; 4 Centro per l’Eccellenza Clinica, la Qualità e la Sicurezza delle Cure (CNEC), Istituto Superiore di Sanità, 00118 Roma, Italy; marco.dinitto@iss.it; 5 Department of Nursing and Obstetrics, Division of Family and Pediatric Nursing, Wroclaw Medical University, 51-618 Wroclaw, Poland; anna.rozensztrauch@umw.edu.pl

For the celebration of International Nurses Day in 2022, the International Council of Nurses (ICN) has proposed the slogan “Nursing, a voice to lead: Invest in nurses and respect rights for global health” [1]. There can be no nobler goal for a modern and transformative health system, especially for transforming nurses, than to uphold the rights of citizens to improve global health, i.e., planetary health. For example, it is obvious and well-known that disease is not distributed randomly among people; therefore, getting sick is not totally a matter of bad luck but rather of where you were born, where you live, what habits you have acquired in your living environment, and what resources you have, and nurses are a valuable resource to combat health inequities [2]. Additionally, for this reason, in a globalized, rarefied, and warlike world, promoting a new, more agile, decisive, diverse, and sustainable health care model should be a priority. Moreover, in this changed model, nurses must be part of the solution and not spectators. Furthermore, for exactly this reason, nursing must be a voice to lead, and not a voice to accompany, because the change in the health care model will be accomplished by the nurses, or it will not be accomplished at all. There is thus a fascinating challenge that nurses face in order to continue to grow as professionals and to rise to the need and challenge. It is convenient to share our particular vision of the present and the future of nursing and of the need for more nursing leadership, greater proactivity towards the health system and the transformation of the care model, and more participation of nurses in all the essential forums, especially in the decision-making that affects not only nurses, but also the entire population. The World Health Organization has long insisted on the need for more nurses to participate in health policies, although this has been reaffirmed and made more visible since 2020 with the Nursing Now campaign [3]. Worldwide, more nurses are necessary in decision-making that translates into a true and more powerful use of advocacy for health. Empowerment and leadership are key: particularly with nurses in active decision-making, it has been found that certain nursing care circumstances are objectively excellent in influencing health outcomes, with decreased morbidity and mortality, increased quality and improvement of care, and cost containment, along with multiple other benefits for society, citizens, users and professionals., All of this is possible with the influence of health advocacy [4].

Advocacy for health will allow what we propose to be exercised. Health professionals are responsible for being guarantors for the advocacy for the population’s health and for watching over the interests of patients and their families and respecting their will [5]. Advocacy towards the nurses themselves is also needed, as health professionals must become guarantors and watch over their interests, since the interests of the nurses are those of the population itself. For example, according to “The triple impact” report, if more is promoted and invested in nursing, three direct results will undoubtedly be obtained: first, improvements in clinical care and in patient and population care; second, reduction and improvement in gender inequality, causing greater equity; and third, the promotion of the sustainability of health systems by improving the countries’ economies [6]. Leadership seems to be a key element in what the ICN proposes and the WHO endorses. In higher nursing curricula, conceptual understanding and competence in leadership have been intensively worked on for years. It is also common to find postgraduate courses focused on leadership and management. We are training future nurses to encourage proactive attitudes. However, more recently, the concept of Political Competence has been incorporated, from very different perspectives, when working with nursing students. As Rosa María Alberdi [7] argues, it is essential that nurses assume their disciplinary and social responsibility and participate in politics, although not necessarily as professional politicians (although perhaps also as professional politicians, since each political decision that is made affects the health of the population for better or worse).

However, at a minimum, nurses must exercise political competence as the health agents that they are, with the ability to transform society as the ICN demands, so that the rights of citizens are respected and that planetary health is preserved and improved. Additionally, to do so, it is necessary to act and work close to where health decisions are made, e.g., in urban planning, transport, waste, housing, work, economy, and commerce. Moreover, nurses are health professionals with an excellent qualifications to participate and intervene in public life, given their versatility and experience in working with the community. Alberdi maintains that this should not be a job, but a commitment, a priority in the social framework, which, according to our vision, expands the multiple commitments that nurses, due to their disciplinary idiosyncrasy, already have with society.

Many more nurses are needed. The nursing workforce is deficient, and the hiring of more nurses needs to be prioritized politically, as practically all relevant institutions claim, and as the ICN and the WHO Nursing Now campaign have maintained [6].

However, more nurses are needed to whom the health system allows a greater development of skills without corporate resistance or private interests. In particular, leading nurses are needed who practice transformative and friendly leadership capable of breaking classic, outdated, and obsolete models of care based on hierarchical and ineffective work teams, i.e., those that claim that “We have always done it that way here and it works well for us”. This is needed to transform reality by encouraging creativity and innovation and incorporating new and good practices, research, community care and public health, which inspire leaders in areas beyond direct health care.

This transformational leadership is what the nursing of the future needs. We are convinced that the health sector and society share the need for more nurses and for a stronger, more active, more participatory and more transformative nursing leadership, which is in all the forums where it should be: at the hospital level, in primary care, in socio-health centers, in public health, at the local level, in schools, at work, in politics, and in research [8]. These nurses must be able to participate in the decision-making that affects not only nurses but also the community. We assume that the environment presents resistance to change in order to try to preserve unnecessary and selfish power hierarchies; this is another type of inequality that, like all inequalities, is unfair, unnecessary, and avoidable.

However, in the end, it is only about putting the patient and their family at the center of the system and making the most of what each professional can contribute, including nurses, specialist nurses, and advanced practice nurses.

Nurses have plenty of knowledge, talent, competence, and responsibility. They have everything needed to help make health systems better, from leadership to nursing competence. Let us empower ourselves as nurses to transform social reality and improve the health level of the population.

## References

[1] International Nurses Day. https://www.icn.ch/what-we-do/campaigns/international-nurses-day.

[2] Phillips J., Richard A., Mayer K.M., Shilkaitis M., Fogg L.F., Vondracek H. (2020). Integrating the Social Determinants of Health into Nursing Practice: Nurses’ Perspectives. J. Nurs. Scholarsh..

[3] Zabalegui A. (2020). Nursing Now. Rev. Cient. Soc. Esp. Enferm. Neurol..

[4] Kelly P.J. (2018). Nurses and global health. Public Health Nurs..

[5] Gea-Caballero V., Castro-Sánchez E., Juárez-Vela R., Sarabia-Cobo C., Díaz-Herrera M.A., Martínez-Riera J.R. (2018). Entorno de práctica profesional en enfermería. Rev. Panam. Salud Publica.

[6] Nigel Crisp L., Watkins of Tavistock B.M. (2018). The triple impact of nursing. Int. J. Nurs. Stud..

[7] Alberdi Castell R. (2019). La competencia política enfermera. Rev. ROL Enferm..

[8] Landa Rivera R.A., Gea-Caballero V., Martínez Riera J.R. (2022). Especial Enfermería Comunitaria Mexico 2022. Editor. RIdEC.

